# Nucleus–Mitochondria Contact Sites Are Associated With Asynthetic Fission in Zebrafish Skin

**DOI:** 10.1177/25152564241239445

**Published:** 2024-03-14

**Authors:** Dhani Tracey-White, Matthew J Hayes

**Affiliations:** Institute of Ophthalmology, 4919 UCL, London, UK

**Keywords:** nucleus–mitochondria, membrane contact site, contact site, asynthetic fission, danio rerio, epidermis, site of membrane contact, corneocyte, keratocyte

## Abstract

Rapid increase in body surface area of growing zebrafish larvae (*Danio rario*) is partially accomplished by asynthetic fission of superficial epithelial cells (SECs) of the skin. There are two cycles of this atypical form of cell division which is unaccompanied by DNA replication; resulting in cells with a variable DNA content. Here, electron microscopy of basal epithelium cells that give rise to these SECs in zebrafish larvae shows aggregation of mitochondria around the nucleus and the formation of nucleus–mitochondria membrane contact sites. Membrane aggregates appear in the lumen of the nuclear envelope at these sites of membrane contact in some cells, suggesting lipid turnover in this vicinity. As the epithelial cells mature and stratify, the mitochondria are engulfed by extensions arising from the nuclear envelope. The mitochondrial outer membrane fragments and mitochondria fuse with the nuclear envelope and parts of the endoplasmic reticulum. Other organelles, including the Golgi apparatus, progressively localize to a central region of the cell and lose their integrity. Thus, asynthetic fission is accompanied by an atypical pattern of organelle destruction and a prelude to this is the formation of nucleus–mitochondria membrane contact sites.

## Introduction

Formation of the cornified, stratified, squamous epithelium of mammalian skin is a well-known process organized into columns of cells called epidermal proliferation units. Basal epithelial stem cells divide symmetrically to form more stem cells, or asymmetrically, to self-renew and generate so-called ‘transit-amplifying cells’ that undergo further rounds of division or differentiation. Loss of the outer skin layer ‘desquamation’, accompanied by proliferation from inner layers, results in the transit of cells through the epidermis. During this transit, the cells alter their expression profiles, commit to differentiation, and withdraw from the cell cycle. These cells eventually become corneocytes in the outermost layer of the epidermis, the stratum corneum (Lechler and Fuchs, 2005). At this stage, the corneocytes have lost all their organelles including their nuclei. This process is associated with production of the structural protein loricrin, which can comprise more than 70% of the cornified cell membrane and significant keratinization (Mehrel et al., 1990).

The epidermis of the model organism zebrafish is somewhat different to that of mammals. Keratinization does not progress as far as it does in mammals. The outermost cells, called superficial epithelial cells (SECs) are crowded with microfilaments that provide strength to this protective later but do not express loricrin. Cells in the intermediate layers have fewer filaments and include a reservoir of undifferentiated cells that can divide in response to damage to the outer layers (Quilhac and Sire, 1999). The inner-most layer of the epidermis is formed of basal epithelial cells (BECs) strongly attached to a basal membrane via hemidesmosomes.

While the skin of adult fish is largely covered by scales that form from the dermis 30 days post-fertilization, the skin of larval zebrafish is fully exposed and has a simple architecture, being composed of the basal layer, an outer SEC layer and, depending on where on the body the skin is examined, a few intervening layers (zero, one or two in larvae < 9 days post-fertilization). Due to the similarity of zebrafish larval skin to mammalian skin, it is a useful model for human skin diseases (Li et al., 2011).

By following the fate of individual SECs in the skin of zebrafish larvae Chan et al. identified a novel form of cell division, which they termed *asynthetic fission*, in which the SECs divide twice without replicating their DNA (Chan et al., 2022). The division of nuclear material is haphazard resulting in four terminally differentiated cells with a variable amount of DNA. Although the reason for this atypical form of cell division is unclear, it is sufficiently unique that it has been characterized, along with mitosis and meiosis, as only the third form of cell division identified to date.

Inter-organelle sites of membrane contact have been identified between nearly all classes of organelle and are involved in the transport and exchange of small molecules and lipids (reviewed (Elbaz and Schuldiner, 2011; Gatta and Levine, 2017; Prinz et al., 2020)). It is likely that all organelles form contact sites with the endoplasmic reticulum and those between the endoplasmic reticulum and mitochondria have been well characterized (Csordás et al., 2018; Wu et al., 2018; Vecellio Reane et al., 2020; Aoyama-Ishiwatari and Hirabayashi, 2021). Those sites of membrane contact between the nuclear envelope, a specialized region of the endoplasmic reticulum, and mitochondria, have only recently been characterized in detail (Desai et al., 2020; Eisenberg-Bord et al., 2021; Ovciarikova et al., 2022; Zervopoulos et al., 2022).

Here, using electron microscopy, we identified clusters of mitochondria around the nucleus in the BECs and superficial epithelial cells in the epidermis of larval zebrafish skin. Close examination of serial sections of these clusters revealed nucleus–mitochondria sites of membrane contact. By comparing cells at different stages of maturity, we find that the formation of nucleus–mitochondria contact sites in these cells precedes a process of organelle destruction and loss with similarities to squame formation in mammals.

## Results

### Nucleus–Mitochondria Contact Sites in the Larval Zebrafish Skin

**Figure 1.**
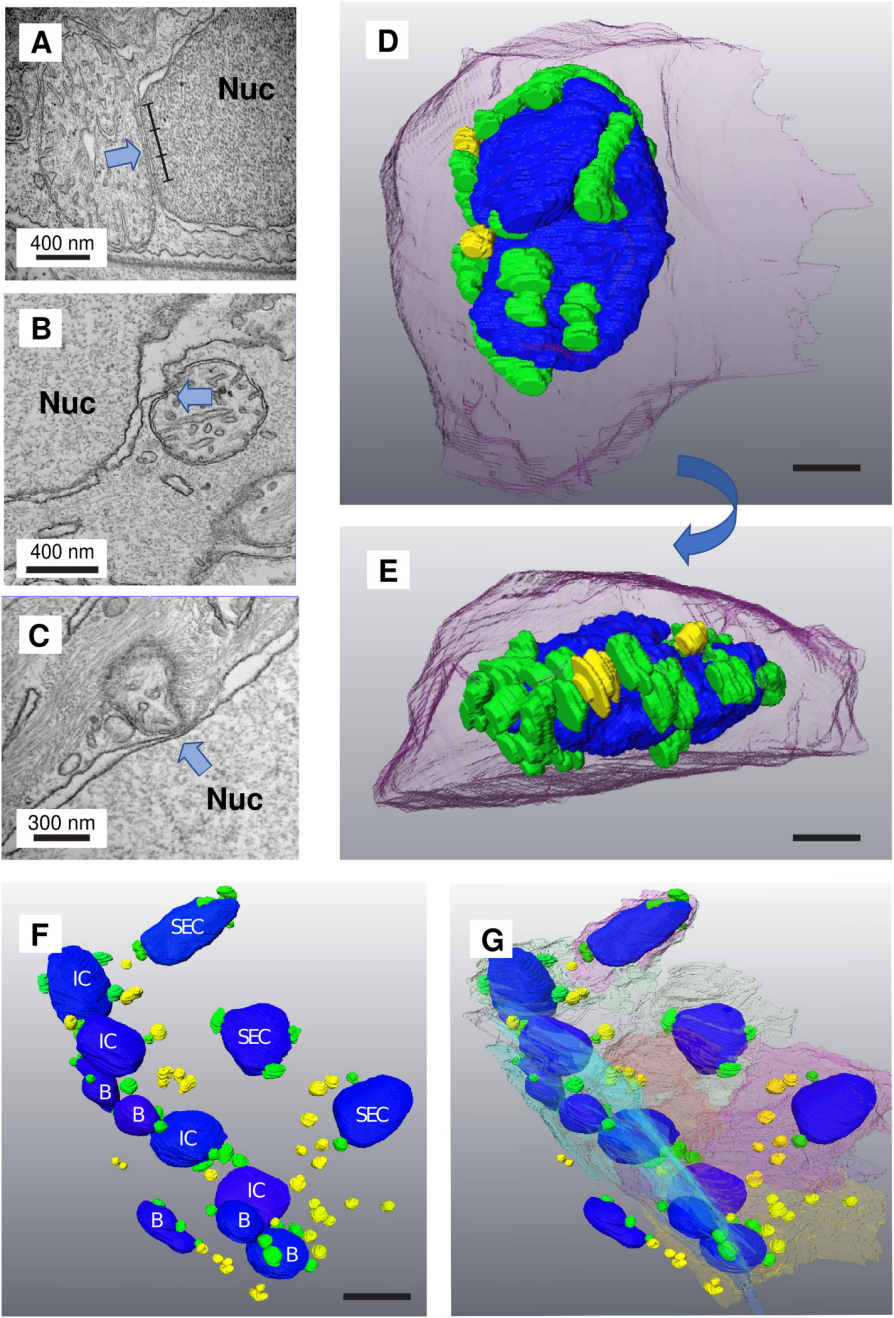
Mitochondria aggregate around the nucleus in epithelial cells in zebrafish and form nucleus–mitochondria sites of membrane contact (images from 4 to 6 dpf). Mitochondria were seen to cluster around the nucleus in 3, 4, 5, and 6 day larval zebrafish epithelial cells. (A–C) TEM images showing mitochondria in close apposition with the nuclear envelope. The contact site is indicated with the blue arrows. The ruler marks a 600 nm contact site. (D) 3D segmentation from SBF-SEM showing a basal epithelial cell (BEC) with mitochondria clustered around it (nucleus in blue, contacting mitochondria green, non-contacting mitochondria in yellow, cell membrane (transparent magenta). Scale bar = 1 µM. (E) A side-on view of the same cell showing the flattened basal surface and aggregation of mitochondria. (F) A region of skin in the vicinity of the larval zebrafish cheek showing several layers of cells: BECs (B), superficial epithelial cells (SEC), and intermediate cells that are found between these two layers (IC). (G) In this image, the cell membranes have been segmented to give an idea of the overall cell shape. Scale bar = 5 µM.

**Figure 2.**
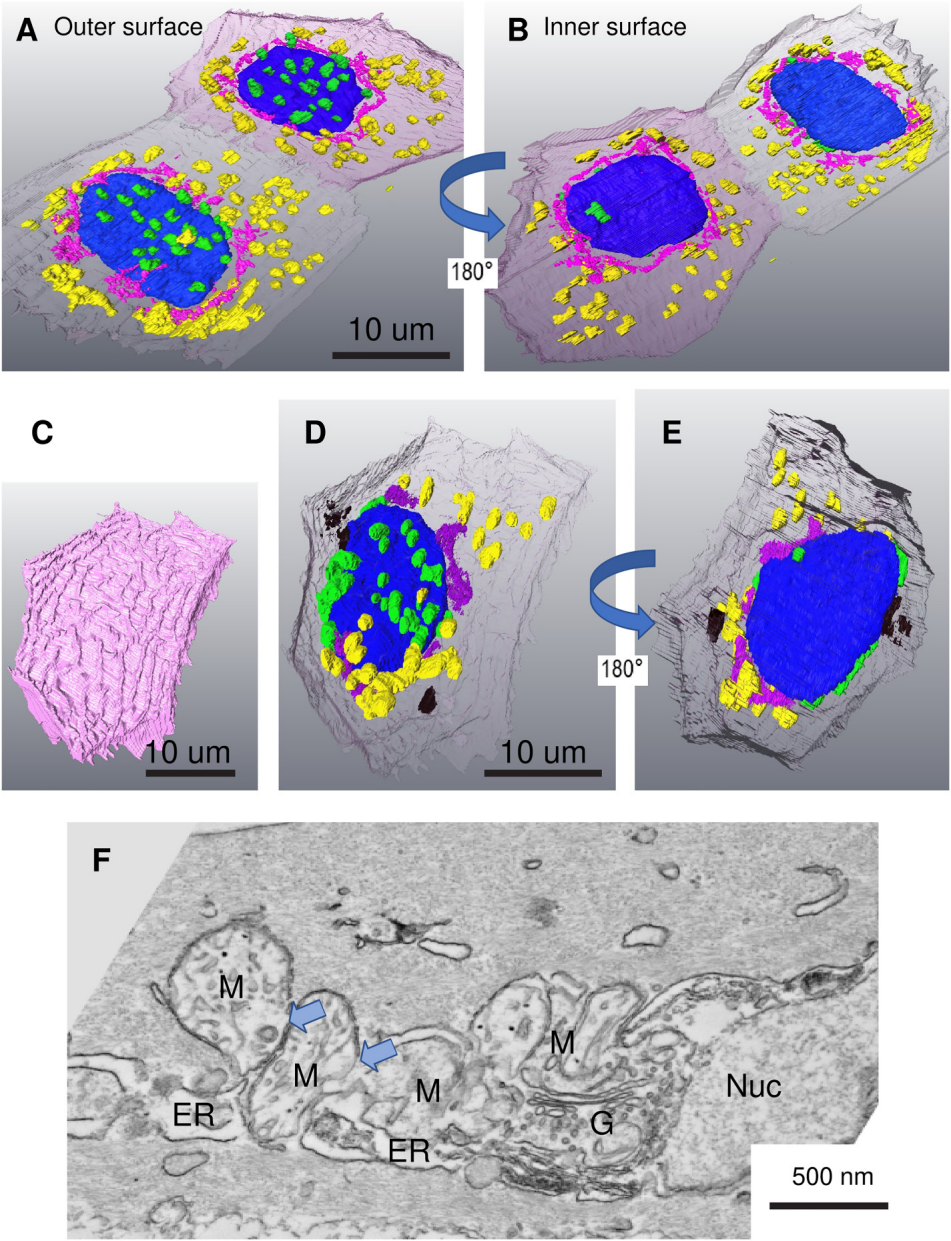
Aggregation and fusion of mitochondria on the surface of the nucleus. (A–B) Segmentation of SBF-SEM images form a 3 dpf zebrafish larva showing the very thin epithelium covering the developing eye. These cells are just beneath the outer layer of mature SECs. Mitochondria in contact with the nucleus are almost exclusively found on the outer surface of the nucleus. Unattached mitochondria are distributed peripherally, often in aggregates, positioned around an elaborate Golgi. (A) The outer-facing surface of two SECs, (B) the inner-facing (showing the underside of the nucleus). Contacting mitochondria (green), non-contacting mitochondria (yellow), nucleus (blue), Golgi (pink) (C) The outer membrane of a mature SEC showing the characteristic ridges. (D) In this mature SEC, the tethered mitochondria are still attached on the outer surface of the nucleus but have begun to aggregate (shown in black). Unattached mitochondria and Golgi appear to have aggregated. (E) The same cell viewing the inner-facing surface. (F) Conventional TEM image showing mitochondrial aggregation and fusion close to the nucleus. Pale blue arrows indicate sites where the outer mitochondria membrane is lost. Nucleus (nuc), mitochondria (M), Golgi (G), endoplasmic reticulum (ER).

Skin was examined from the face, flank, fins, and tail of zebrafish larvae (3, 4, 5, and 6 dpf) by transmission electron microscopy (TEM). In basal epithelial cells (BECs) (identified as those attached to the basal membrane), in all areas examined and at all ages, mitochondria appeared closely clustered around the nucleus (Figures 1 and 2). In transverse sections, the BECs have a flattened appearance with a lenticular nucleus. Quantitation was performed on 5dpf larvae (n  =  5) by examining at least 25 cells picked at random in the skin on the surface of the flank. We only included cells which were sectioned near the centre of the nucleus and mitochondria were considered ‘clustered’ if they were less than 200 nm from the nuclear envelope. 60 ± 4.2% (n  =  5 5dpf larvae) of mitochondria were within this zone. Approximating the cell and cell nucleus to be a flattened oblate spheroid, this 200 nm zone is less than 5% of the cell volume. At some sites, the mitochondria were so closely opposed to the nuclear envelope that the gap between the outer membrane of the mitochondria and the nuclear envelope was less than that between the inner and outer membranes of the mitochondrion (approximately 20 nm) (Figure 1). We identified cells in which the nuclear envelope appeared ‘drawn out’ by this contact, accompanied by an increased lumen between the inner and outer leaflets of the nuclear envelope in the region of contact (Figure 1B). This suggests a covalent attachment between the mitochondrion and the nucleus. In other places the mitochondria seemed to have pushed the leaflets together, appearing to be embedded in the surface of the nucleus (Figure 1C). These may be described as nucleus–mitochondria sites of membrane contact, something that has been described in detail in budding yeast (Eisenberg-Bord et al., 2021) and in several cell lines in response to stress or proliferative stimuli (Desai et al., 2020; Zervopoulos et al., 2022). Of 125 cells it was possible to measure the extent of the potential contact site in 50 cases. The mean length was 263 nm ± 190 nm.

The TEM data from individual sections suggested that mitochondria clustering might be due to limited, partial contacts between the mitochondria and the nuclear envelope. We used serial block face scanning electron microscopy (SBF-SEM) to generate a stack of images through the head of 5dpf larvae (n  =  3). Briefly, SBF-SEM utilizes a diamond knife microtome mounted inside a conventional SEM which removes sections from a resin block containing the heavy metal-stained specimen. The specimen is imaged using a tuned detector of back-scattered electrons from the scanning beam that are emitted from the surface of the polished block following interactions with these heavy metals. Repeated, serial sectioning and subsequent imaging, generate a large stack of images (potentially several thousand) separated by the thickness of the removed section 70 nm in this case). Segmentation of these SBF-SEM image stacks of skin from these larvae and subsequent 3D reconstruction revealed that clustered mitochondria make intimate contacts only along part of their surface, a feature that is not always apparent in single, thin 2D (50 nm) TEM sections. In some cells most of the mitochondria are clustered around the nucleus, in others, less than half are associated with the nucleus (Figure 1D–G, Figure 2A–E). SBF-SEM has a resolution of 13 nm in these images, which is sufficient to observe true contact sites.

Analysis of the 3D reconstructions revealed no statistically significant difference in the length or width of mitochondria contacting the nucleus compared to those un-tethered in the cytosol (data not shown). In the very thin BECs overlaying the developing eye, nucleus–mitochondria contacts were only observed on the outward facing surface of the flattened nucleus, the inner face being almost devoid of such contacts (97.2% on the outer face ± 2.3%, n  =  5 cells). Non-contacting mitochondria encircle the nucleus, many in association with extensive Golgi (Figure 2A–E).

### Organelles in the Outermost SECs Undergo Aggregation

**Figure 3.**
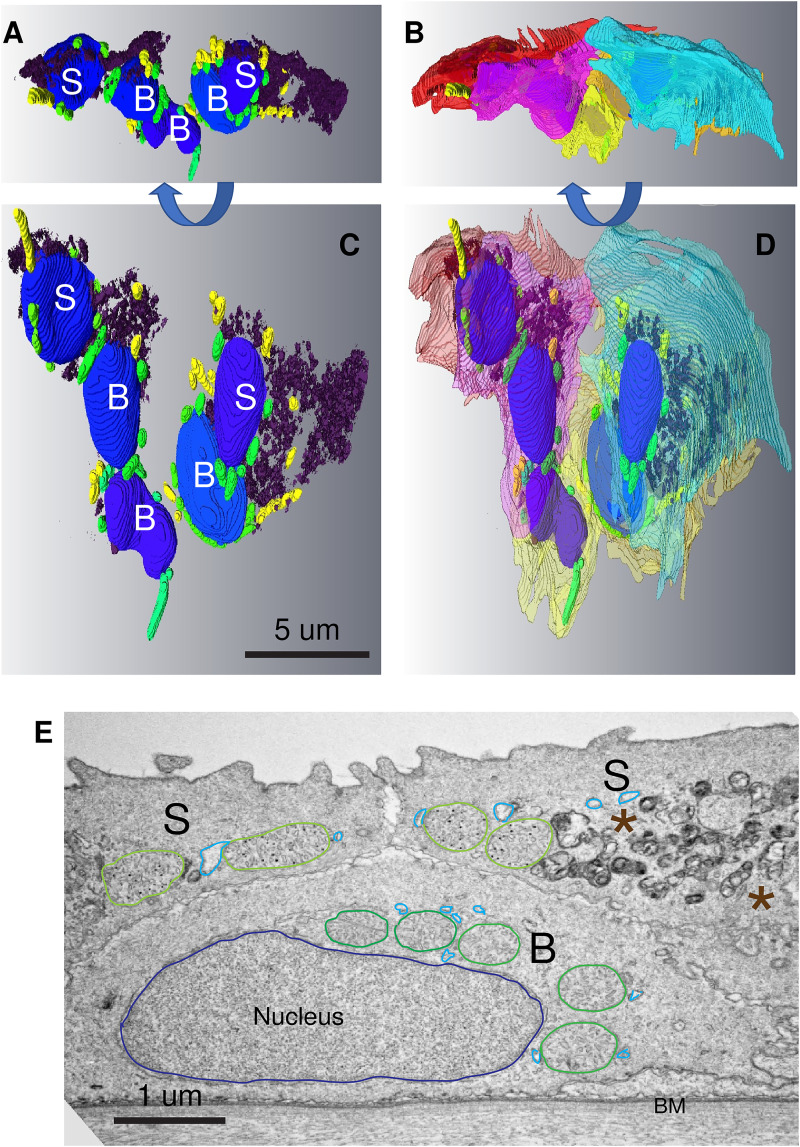
In 6 dpf SECs mitochondria and Golgi apparatus, embedded in endoplasmic reticulum, undergo disintegration. (A and C) Segmentation of a group of cells in the vicinity of the eye in a similar orientation to the TEM image shown in A. The electron-dense material has been automatically segmented out and is mostly associated with the mature SECs on the outside of the cells. (B and D) 90° rotations of A and C to better reveal the arrangement of mitochondria and debris. Nuclei (blue), tethered mitochondria (green), un-tethered mitochondria (yellow), electron-dense debris (dark purple), endoplasmic reticulum (pale blue) SECs (S), BECs (B), basal membrane (BM). (E) Conventional transverse TEM image of skin in the vicinity of the developing eye of a 6-day-old larva. Mitochondria (outlined in dark green) can be seen clustered around the nucleus in the basal cell (B). In mature SECs (S), the mitochondria (outlined in pale green) have begun to disintegrate and much electron-dense organelle debris has accumulated (asterisks).

In our samples it was impossible to expressly establish the age of any individual cell; but we can assume that basal cells, in contact with the basement membrane, are younger than those in the outer layers of the skin. Where more than one layer exists, it is likely that the younger cells are closer to the basal cells from which they are derived. Examination of SECs (classified as those cells with extensive ridges on the outermost surface of the skin (Figure 2C) by SBF-SEM), revealed the redistribution of organelles. Mitochondria appeared as aggregates close to the nuclei and the Golgi appeared more densely packed and less distributed throughout the cell compared to cells at lower layers of the epidermis (Figure 2A and 2F, Figure 3B–E). By conventional TEM the organelles in some cells could be seen to have undergone loss of integrity and appeared as electron-dense aggregates (Figure 3A). This suggests generalized loss of integrity of the organelles as the cells age.

### The Nuclear Envelope Engulfs Mitochondria at Nucleus–Mitochondria Sites of Membrane Contact

**Figure 4.**
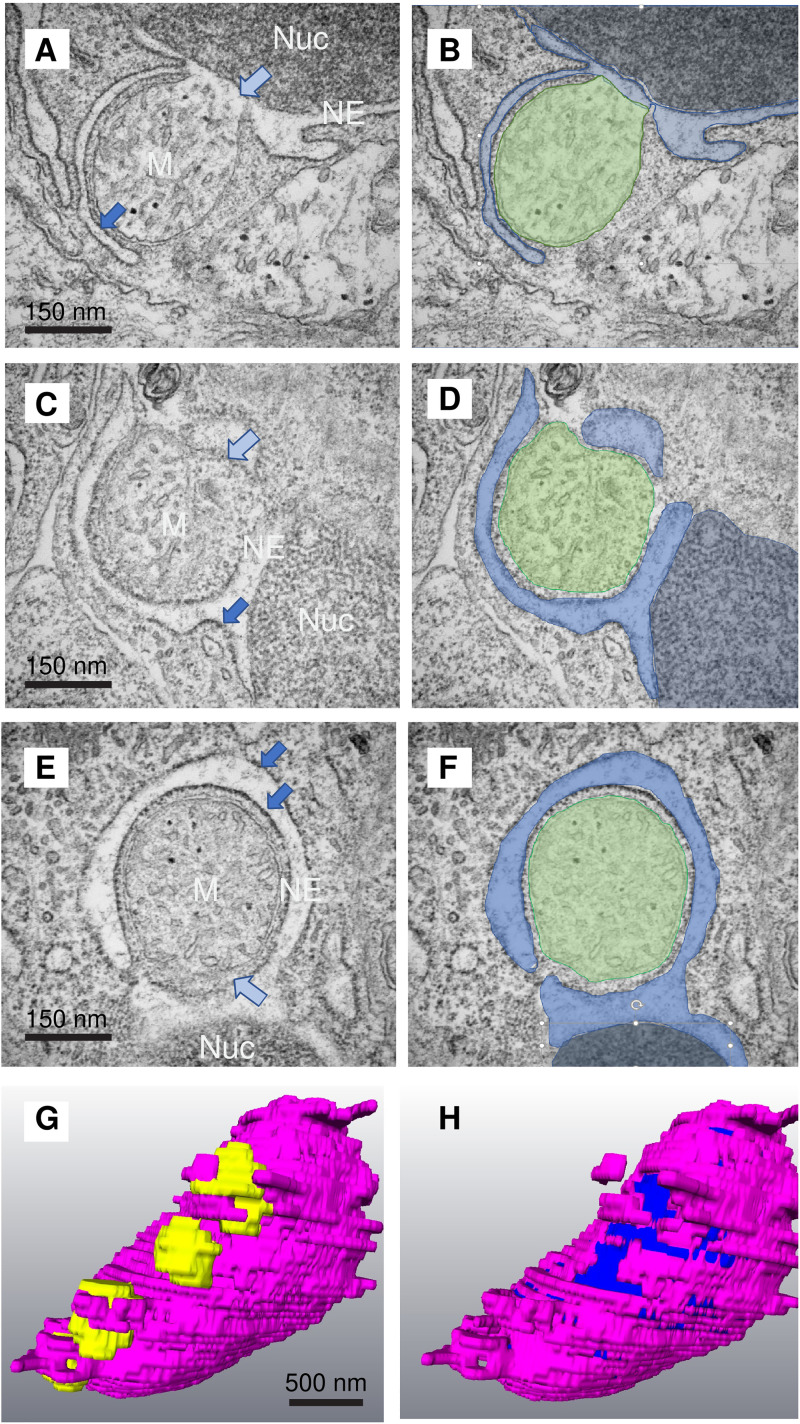
Mitochondria are engulfed by the nuclear envelope at the site of nucleus–mitochondria contact. (A–F) Nuclear envelope (endoplasmic reticulum) extended around the contacting mitochondria in 5dpf fish. Nucleus (Nuc: dark blue), mitochondrion (M: green), nuclear envelope (NE: pale blue). The outer mitochondrial membrane was often ruptured on the face of the mitochondrion closest to the nucleus (pale blue arrows). Sometimes the endoplasmic reticulum extensions were heavily decorated with ribosomes (dark blue arrows). G: Segmentation of an SBF-SEM stack from a 3 dpf zebrafish larva showing mitochondria (yellow) surrounded by ER extensions arising from the nuclear envelope. H: As G but with the mitochondria removed revealing the exposed nuclear matrix lying beneath the sites of mitochondrial engulfment (dark blue).

Examination of fields of epithelial cells in the skin by TEM revealed numerous examples of mitochondria juxtaposing the nuclear envelope which were also closely associated with other regions of endoplasmic reticulum. In some cells, we see that at least some of these are extensions of the nuclear envelope arise very close to the site of nuclear–mitochondrial contact (Figure 4A–H). These extensions may be of smooth membrane (smooth ER) or studded with ribosomes (rough ER). In some cases, the nuclear envelope appears to engulf the mitochondrion by means of an extended mitochondria–ER contact. Such contacts are reminiscent of autophagosomal membranes) and of MAMs (smooth mitochondria-associated membranes and wrappER (sterol-rich rough ER wrapped around mitochondria) observed in other systems (Anastasia et al., 2021). Close examination of these sites revealed that the mitochondrial outer limiting membrane is sometimes missing in the region closest to the nucleus and we often observe fragmentation of the nuclear envelope itself. Figure 1A shows a 600 nm nucleus–mitochondria contact site with a 400 nm region in which only 3 membranes are visible.

### Accumulation of Electron-Dense Aggregates in Mitochondria and in the Nuclear Envelope Lumen

**Figure 5.**
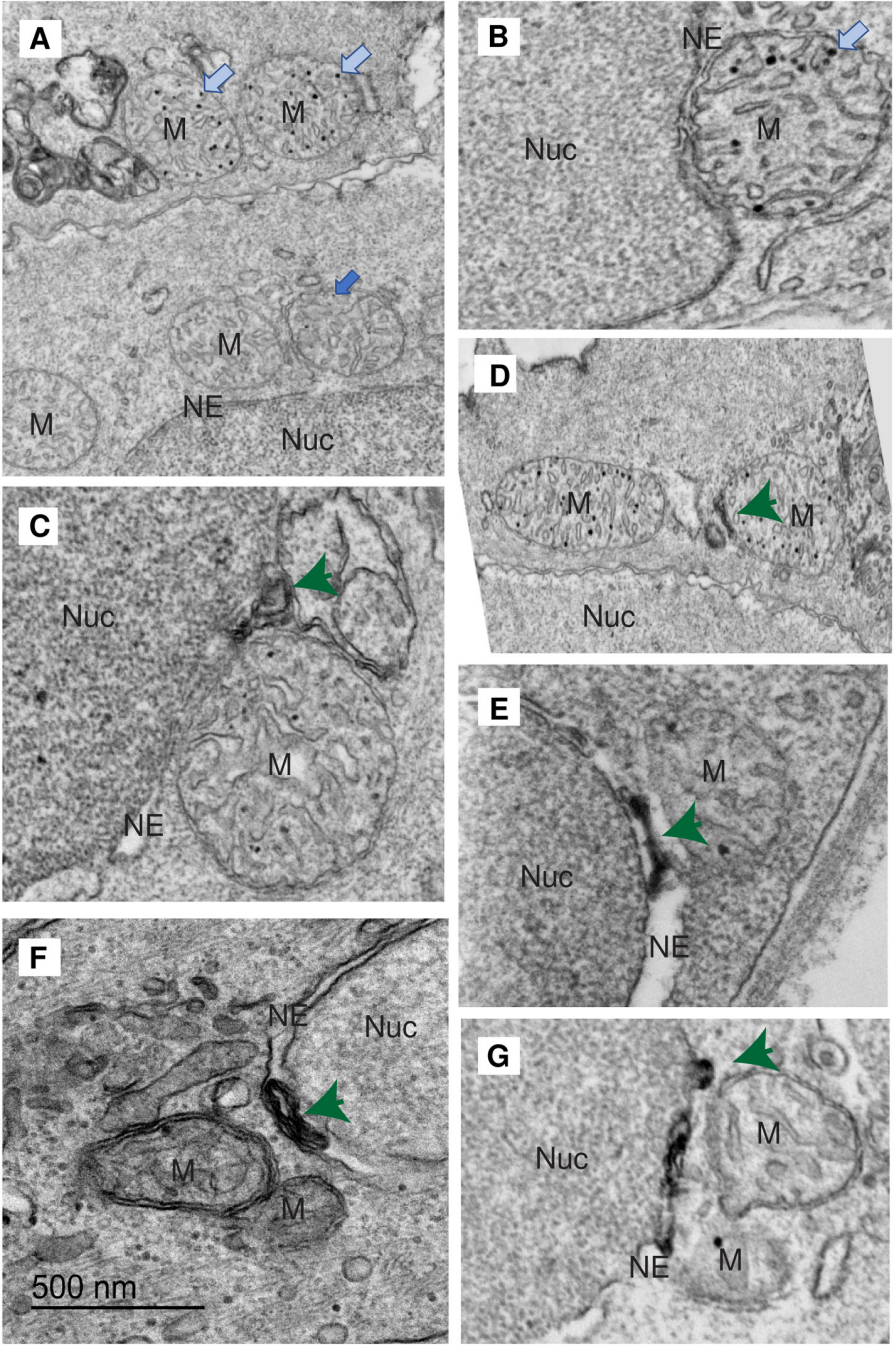
Mitochondria and the ER lumen juxtaposed to mitochondria-nuclear sites of membrane contact in mature SECs from 5 and 6 dpf animals contain electron-dense inclusions. (A, B) Mitochondria in (M) clustered around the basal cell nucleus (Nuc) have few internal lipid droplets (electron-dense puncta dark blue arrow). Mitochondria in mature SECs have many (pale blue arrows). (C–G) We often observed accumulation of electron-dense staining (mostly likely lipid in these preparations) in the lumen of the nuclear envelope (NE) in the vicinity of the contacting mitochondria (dark green arrows). Note in C and D the partial loss of the mitochondrion outer membrane. In some cells, this was entirely missing from the mitochondria.

During the processing of TEM samples, osmication results in electron-dense staining of lipids. Mitochondria in SECs contain electron dense, round particles in their matrix (Figure 5A–D). At sites of nucleus–mitochondria membrane contact, we also observed electron-dense aggregates (Figure 5E and G) and membranes, sometimes within the lumen of the nuclear envelope in the vicinity of the contact site (Figure 5C and F) suggesting the accumulation of lipids at these sites.

## Discussion

In mammals corneocyte maturation involves the complete destruction of the nucleus (Matsui et al., 2021) and all organelles. In this study of larval fish epithelium, we observe abundant nucleus–mitochondria sites of membrane contact, partial breakdown of the nuclear and mitochondrial membranes and general disorganization of the organelles. The nucleus, however, remains intact. Instead of initiating autophagy and apoptosis, the cell survives and is not completely sealed off from its neighbours as is the case in the mammalian epidermis.

Nucleus–mitochondrial contact sites have been implicated in anterograde and retrograde trafficking of both survival and apoptosis-promoting molecules (reviewed in (Walker and Moraes, 2022)). It is tempting to speculate that the nuclear-mitochondrial sites of membrane contact could provide a mechanism for the survival and proliferation of SECs, in spite of the partial loss of organelles. Nucleus–mitochondria sites of membrane contact formed by mitofusin-2 tethers have been described in synchronized A549 cells (human lung adenocarcinoma cells) in response to serum plus epidermal growth factor (EGF) and in HFF-1 cells (human foreskin fibroblasts) in response to hypoxia; both of which induce proliferation (Zervopoulos et al., 2022). This process is thought to explain the non-canonical nuclear entry of the mitochondrial pyruvate dehydrogenase complex (PDH), a megacomplex far too big to enter via the nuclear pores. Provision of this enzyme is necessary for production of acetyl-CoA leading to acetylation of histones, an epigenetic modification of chromatin which usually favours transcription (Grunstein, 1997). The authors suggest partial breakdown of the nuclear envelope and formation of mitochondria-derived vesicles as a means for the PDH to exit the mitochondria and enter the nucleus; but do not provide direct evidence for this. The partial membrane fusions we observe in our study could provide such a mechanism; but it remains to be shown whether such membrane fusions are conserved into mammals or whether PDH enters the nucleus in larval fish SECs.

An important role for nucleus–mitochondria sites of membrane contact has also been established in the mitochondria retrograde response (MRR); in which stressed or damaged mitochondria communicate with the nucleus. In an in vitro model for breast cancer (MCF-7 and MDA-MB-231 cells) the ATP-competitive kinase inhibitor staurosporine caused clustering of mitochondria around the nucleus. A contact site is formed between the outer mitochondrial membrane protein TSPO and ACBD3 a protein recruited to the nuclear envelope by association with PKA and AKAP95. This results in the nuclear translocation of the pro-survival transcriptional factor NF-kappaB. Mouse embryonic fibroblasts over-expressing TSPO coalesce the mitochondria with the nucleus, accumulate NF-kappaB in their nuclei and became resistant to staurosporin induced apoptosis. Interestingly, knockdown of MFN2 had little effect in this system. (Desai et al., 2020). It therefore appears that there may be more than one tether that maintains nucleus–mitochondria contact sites. The trafficking of pro-survival signals from the mitochondria to the nucleus in the fish epidermis could explain how SECs avoid apoptosis. This needs further examination

Exchange of lipids, including cholesterol, ceramide, phosphatidyl serine, phosphatidyl ethanolamine and phosphatidyl choline have all been described at mitochondria-ER contacts (Reinisch and Prinz, 2021). Osmication of unsaturated phospholipids results in electron-dense staining in electron micrographs. Our observation of accumulation of membranous electron-dense structures at the nucleus–mitochondrial site of membrane contact could represent accumulation of lipids at this site. However, harder to explain, is how the material has transferred to the nuclear-envelope lumen. Unfortunately, the SBF-SEM data was of insufficiently high resolution to see if the membranes inside the nuclear envelope lumen are continuous with the nuclear envelope or completely internalized.

The extensions from the nuclear envelope we observe ‘engulfing’ the mitochondria are reminiscent of autophagosome membranes observed in mitophagy. Contact sites between the mitochondrion and the endoplasmic reticulum have been shown to be the sites of autophagosome formation (Hamasaki et al., 2013). In yeast, mitophagy is thought to be initiated at contacts mediated by the ER–mitochondria encounter structure (ERMES) which facilitates phospholipid transfer between mitochondrion and ER (Böckler and Westermann, 2014). In mammalian cells, the ER, Golgi, plasma membrane, endosomes, and even the mitochondria themselves, have all been posited as potential sources of lipids that contribute to the autophagosome membrane (Ktistakis, 2020), (Ktistakis, 2021). Indeed, at the early stages of autophagosome formation, the so-called omegasome is often associated with the endoplasmic reticulum. It is quite possible that our observations represent a form of mitophagy; but there are several deviations from canonical observations. The surrounding membranes remain attached to the nuclear envelope and are not disengaged. We have also observed rough endoplasmic reticulum membranes (with embedded ribosomes) in these structures and canonical autophagosome membranes are devoid of protein markers of their membrane of origin. The complex membrane fusions we observe are also not something described in classical mitophagy.

In mammalian skin the keratinocytes completely lose their organelles during cornification (Eckhart et al., 2013) (Lavker and Gedeon Matoltsy, 1970). We speculate that the situation in larval fish is an intermediate one in which senescence and cellular death is initiated but the cells are ‘rescued’ by the nucleus–mitochondria contacts and survive long enough to undergo additional rounds of asynthetic cell division (Chan et al., 2022).

## Methods

### Zebrafish Husbandry

The wild-type AB zebrafish embryos were kindly gifted by UCL zebrafish facility. Adult zebrafish (wild-type, AB strain) were bred and maintained according to UCL and UK Home Office regulations for the care and use of laboratory animals under the Animals Scientific Procedures Act 1986. All zebrafish embryos were raised at 28.5 °C on a 14-h light/10-h dark cycle and were euthanized at the same time of day. At least 5 zebrafish larvae were examined at each age examined.

### Conventional TEM

Samples were fixed in ½ Karnovsky's fixative. After rinsing in 0.1 M sodium cacodylate-HCl buffer (pH 7.4), the samples were post-fixed in 1% aqueous osmium tetroxide/1% potassium ferrocyanide for 2 h, rinsed with ddH_2_0 then dehydrated with ethanol (1  ×  30%, 50%, 70%, 90% and 2  ×  100%) and then two changes of 100% propylene oxide, and then infiltrated overnight with a 1:1 mixture of propylene oxide: araldite (EPON) on a rotator. Finally, samples were infiltrated with 100% resin over 4–6 h and then embedded in fresh resin, and cured for 48 h at 60°C. On a Leica Ultracut UCT, semi-thin sections (500 nm/0.5 µm) were collected, stained with a 1% toluidine blue-borax, and then imaged on an EVOS Fl Auto 2 system. Ultra-thin sections (75–100 nm/0.075–0.1 µm) were collected on copper TEM grids and were contrasted with Reynold's lead citrate for imaging in a JEOL JEM1400plus TEM. Images were captured using a Deben NanoSprint12S camera using the Advanced Microscopy Techniques (AMT) Imaging Software.

### Serial Block Face Scanning Electron Microscopy (SBF-SEM)

Samples were fixed as above. After rinsing in 0.1 M sodium phosphate buffer (pH 7.4), the samples were post-fixed in 2% aqueous osmium tetroxide/1.5% potassium ferrocyanide for 2 h at RT, then rinsed with _dd_H_2_0. The samples were then placed in 1% thiocarbohydrazide (TCH) solution for 10 min and then rinsed with _dd_H_2_0. A second osmication was performed (2% osmium tetroxide (no ferricyanide)) for 30 min and the samples were then rinsed again with _dd_H_2_0 and then placed in aqueous 2% uranyl acetate overnight at 4°C. The samples were then placed in freshly made Walton's lead aspartate (pH 5.5) for 30 min at 60°C and subsequently rinsed with _dd_H_2_0. Samples were then dehydrated with ethanol (30%, 50%, 70%, 90%) to 100%  ×  3 then in acetone 2  ×  20 min). Samples were then infiltrated with ascending Durcupan resin concentrations (25%, 50%, 75% in acetone) for 2 h each and then left overnight in 100% Durcupan resin. The samples were embedded with fresh resin, and the blocks cured for 48 h at 60°C. The samples were trimmed to 0.75  ×  0.75 mm in size and mounted onto specially prepared aluminium pins using resin. The samples were imaged on a Zeiss Sigma VP Gemini SEM fitted with a Gatan 3 View serial block-face system. Images were analysed and segmented using the Amira Software.
